# Comparison of the burden of diarrhoeal illness among individuals with and without household cisterns in northeast Brazil

**DOI:** 10.1186/1471-2334-13-65

**Published:** 2013-02-04

**Authors:** Pasha B Marcynuk, James A Flint, Jan M Sargeant, Andria Jones-Bitton, Ana M Brito, Carlos F Luna, Elizabeth Szilassy, M Kate Thomas, Tiago M Lapa, Enrique Perez, André M Costa

**Affiliations:** 1 Centre for Food-borne, Environmental and Zoonotic Infectious Diseases, Public Health Agency of Canada, Guelph, Ontario, Canada; 2 Centre for Public Health and Zoonoses and Department of Population Medicine, Ontario Veterinary College, University of Guelph, Guelph, Ontario, Canada; 3 Departamento de Saúde Coletiva-Nesc, Centro de Pesquisas Aggeu Magalhães/Fundação Oswaldo Cruz, Cidade Universitária, Recife, Pernambuco, Brazil; 4 Faculdade de Ciências Me’dicas, Universidade de Pernambuco, Recife, Pernambuco, Brazil; 5 Articulação no Semi Árido, Recife, Pernambuco, Brazil; 6 Pan American Health Organization, PANAFTOSA, Rio de Janeiro, Brazil

## Abstract

**Background:**

Lack of access to safe and secure water is an international issue recognized by the United Nations. To address this problem, the One Million Cisterns Project was initiated in 2001 in Brazil’s semi-arid region to provide a sustainable source of water to households. The objectives of this study were to determine the 30-day period prevalence of diarrhoea in individuals with and without cisterns and determine symptomology, duration of illness and type of health care sought among those with diarrhoea. A subgroup analysis was also conducted among children less than five years old.

**Methods:**

A face-to-face survey was conducted between August 20th and September 20th, 2007 in the Agreste Central Region of Pernambuco State, Brazil. Households with and without a cistern that had at least one child under the age of five years were selected using systematic convenient sampling. Differences in health outcomes between groups were assessed using Pearson’s Chi-squared and two-way t-tests. Demographic variables were tested for univariable associations with diarrhoea using logistic regression with random effects. P-values of 0.05 or less were considered statistically significant.

**Results:**

A total of 3679 people from 774 households were included in the analysis (1863 people from 377 households with cisterns and 1816 people from 397 households without cisterns). People from households with a cistern had a significantly lower 30-day period prevalence of diarrhoea (prevalence = 11.0%; 95% CI 9.5-12.4) than people from households without a cistern (prevalence = 18.2%; 95% CI 16.4-20.0). This significant difference was also found in a subgroup analysis of children under five years old; those children with a cistern had a 30-day period prevalence of 15.6% (95% CI 12.3-18.9) versus 26.7% (95% CI 22.8-30.6) in children without a cistern. There were no significant differences between those people with and without cisterns in terms of the types of symptoms, duration of illness and health care sought for diarrhoea.

**Conclusions:**

Our results indicate that the use of cisterns for drinking water is associated with a decreased occurrence of diarrhoea in this study population. Further research accounting for additional risk factors and preventative factors should be conducted.

## Background

Water is a basic requirement for the healthy functioning of all the world’s ecosystems and is inextricably linked to public health and human development. Although the provision of sufficient, safe, culturally acceptable and accessible water is considered a fundamental human right by the United Nations (UN) [1], the World Health Organization (WHO) estimates that 1.1 billion people lack access to clean water [2]. This is a major contributor to the estimated 4 billion cases of diarrhoea experienced globally each year [3]. Approximately 90% of the 1.8 million deaths per year due to diarrhoea are among children under five years old, mostly in developing countries [4].

The semi-arid region of Brazil is approximately 868,000 km^2^ and is inhabited by over 18 million people [5]. Although average precipitation rates are high compared to other parts of the world, water is scarce due to extremely shallow soils with a low capacity to retain water, unimodal rain patterns, and privatization of the few quality water sources that exist [5]. Rural Brazilian hinterland families in the semi-arid region of Brazil spend as many as 30 hours a month collecting water [Personal Communications, Elizabeth Szilassy]. This task is often delegated to women and children who carry heavy loads of water on their heads, a practice which itself leads to adverse health outcomes such as spinal damage. Assigning children to the role of collecting water also means less time spent pursuing education, further perpetuating the poverty cycle. Furthermore, water sources are often open and thus susceptible to contamination from human, animal, and chemical sources.

Assessments of various water treatment methods that improve water quality, and thus, potentially reduce diarrhoea have been conducted worldwide. Point of use water treatment using filters, solar energy, flocculant-disinfectant, and chlorine, as well as community hygiene education have all been shown to reduce diarrhoea to various degrees [6-17]. However, studies have not been specifically conducted to address the problem in the northeast rural regions of Brazil, where water is not only of poor quality, but is also scarce.

One potential solution to the water quality and scarcity issue is the use of household cisterns to collect rainwater from rooftops. Cisterns that collect and store rainwater from rooftops of family dwellings have been used in rural communities in Southern Australia [18]. In Brazil, the One Million Cisterns Project (P1MC) was implemented with the goal of constructing one million cisterns to collect rainwater from rooftops [19]. This large scale water intervention was launched in 2001 and is supported by the Brazilian government, UN agencies, the Brazilian banking federation and non-governmental organizations (NGOs) such as Oxfam [20]. It is coordinated by Articulação no Semi-Árido (ASA) [19], an umbrella organization that links more than 700 NGOs, farmers’ unions, churches and associations. As of September 2011, P1MC has provided approximately 351,000 cisterns to families throughout the semi-arid region of northeast Brazil [21].

As of 2007 the health impact of the P1MC had not been investigated. Therefore, in August 2007, a survey based study of individuals within households with and without cisterns was undertaken to evaluate the impact of cistern use on various health outcomes. This paper focuses on the impact of household cisterns on the 30-day period prevalence of diarrhoea. The primary objective of this study was to determine the 30-day period prevalence of diarrhoea in all individuals and among children less than five years old with and without cisterns in the Agreste Central Region of Pernambuco State, Brazil—an area within the semi-arid region of north-eastern Brazil. Incidence of diarrhoea was also estimated.

The secondary objective was to determine the symptomology, duration of illness and type of care sought among those who had diarrhoea at least once during the previous 30 days, in households with and without cisterns.

## Methods

A face-to-face survey was conducted between August 20th and September 20th, 2007 in the Agreste Central Region of Pernambuco State, Brazil.

### Study population

The Agreste Central Region of Pernambuco State, Brazil has a total population of 824,441, of which 216,861 are considered rural. This region was selected for the study because it had participated in the P1MC for several years and was planning to build more cisterns in the coming years, providing a sizable population of households with a cistern for more than one year and households without a cistern but eligible for one. Households were eligible for a cistern from P1MC if they were: low income families (defined as an income of less than half of minimum wage), living full time in the rural area, without water security (i.e. did not have a cistern of least a 2,000 L capacity), with one or more children less than five years of age or elderly people (more than 60 years old). Within the eligible families, the community determined which families had a cistern built first. By May 2006, approximately 5,000 cisterns had been constructed in this region.

The Agreste Central Region contains 23 municipalities, with each municipality having between 25 and 250 cisterns already constructed; within each municipality there are dozens of communities. Two municipalities (Bonito and Barra de Guabiraba) were not included in this study as they are located in geographical transition zones in the more humid Zona da Mata, which has a greater availability of water, and because they do not qualify for a cistern from the P1MC. Thus, a total of 21 municipalities and their communities were included in this study.

### Sample size

A total target sample size of 816 households (408 households with cisterns and 408 without) was calculated to detect a difference of at least 10% in the prevalence of diarrhoea in children less than five years, with 95% confidence and 80% power. Children less than five years of age were used to calculate the sample size as local surveillance data has shown that this age group has a higher prevalence of diarrhoea [unpublished data].

### Participant recruitment

Recruitment for participation in the survey was conducted at the household level on the basis of the presence or absence of a cistern. Households with cisterns were only included if the cistern was constructed by P1MC before May 2006, and there was at least one child less than five years old residing in the household. Households were excluded if they were connected to a water system. Random selection of these households was not possible because cisterns were not properly labelled according to a master list of cisterns that had been built in the area by P1MC provided by ASA. Instead, interviewers visited at least two households that had a cistern and met the study criteria in each community (case households) within the 21 municipalities, for the total target of 408 households. The number of households visited in each community was proportional to the number of cisterns that had been built in the community. Households without cisterns were recruited by interviewers who, for every household with a cistern, also visited the next closest home without a cistern provided that they were not connected to a water system and had at least one child under the age of five years. If in each community or municipality there were not enough non-cistern homes to meet the sampling requirement, the next closest community in the municipality (or neighbouring municipality) was selected. This recruitment strategy was used to ensure geographical proximity of households with and without cisterns. Every person in each household was included in the study.

### Survey methods

Surveys were administered in person by 18 local interviewers who were familiar with the communities. To reduce interviewer bias, interviewers were trained prior to data collection using an interactive training session and an interviewer’s manual for reference. In addition, a supervisor was assigned to every four or five interviewers to provide guidance and check interview forms for completeness. Each house was approached up to three occasions during recruitment. If necessary, interviewers returned to the home for additional visits until the survey was complete. During the interview, the date and time for interviewing additional family members not currently available was scheduled. Weekend visits were scheduled where appropriate to accommodate participants. If both heads of household were home, the woman was asked to complete the survey. In the case where neither was home, another adult was approached. The head of the household was asked all questions pertaining to the entire household, and questions regarding their own personal health. Health-related questions for all other residents were asked of each individual. If an individual was under the age of 15 years or an elder (more than 60 years old), the head of the household could decide to respond on their behalf.

The questionnaire was adapted from similar surveys in Canada [22-24] and the Dhanusha Community Drinking Water and Sanitation Project in Nepal (unpublished data) and reviewed and modified for local appropriateness. The questionnaire was translated into Portuguese and pre-testing was conducted in the municipality of Bezerros, where it was administered to five households with cisterns and five households without cisterns (these households were not included in the results). Necessary revisions were made to increase clarity and flow of the questionnaire. On average, questionnaires took 30–40 minutes to complete, depending on the number of family members. All interviews were conducted in Portuguese.

Signed consent was obtained from a head of the household or someone who participated in the management of the household. To maintain confidentiality, paper and electronic copies of the survey were stored in a locked cabinet and encrypted in password protected files on two computers, respectively.

### Survey

This study was part of a larger Household Health Survey with questions pertaining to demographics, health conditions (diarrhoea, respiratory disease, fever, skin conditions, red eye, and intestinal worms), social impact of P1MC, and hygiene behaviours. Households with cisterns were asked additional questions regarding environmental conditions (including roof material, age of cistern, maintenance and condition of the cistern, and mosquito control knowledge and behaviours). The Household Health Survey was conducted to evaluate the effectiveness and impact of the P1MC on health.

The current study focused on the prevalence of diarrhoea. Questions solicited information on the presence or absence of diarrhoea in the 30 days prior to the interview, the number of people residing in the home and demographic characteristics (age, source(s) of income, gender, community name, municipality, self reported literacy of mothers and fathers of the household and, if literate, their highest education level completed). The parents of children under five years old were considered to be the mother and father of the household. Those with diarrhoea in the 30 days prior to the interview were also asked about the number of episodes and days with diarrhoea in the past month, whether their stool was liquidy/watery (a local idiom for cholera) and/or had blood, which medications or home remedies were taken, and whether health care was sought. For certain questions (e.g., sources of income and health care sought), respondents could select more than one response within a category and were represented as binary variables.

### Case definition

The case definition used for acute diarrhoea was diarrhoea with or without vomiting (3 or more loose/liquid stools) in a 24-hour period that was not due to a long-term illness, medication, overindulgence of alcohol or related to pregnancy. This definition is consistent with the WHO’s definition of diarrhoea [25], allowing for comparison with similar international studies. A new episode was considered when there were at least two days without diarrhoea between the two episodes during the 30-day period.

### Analysis

#### Data management

Survey responses were recorded on paper at the homes of the participants, checked for omissions and incorrect data entry by the supervisors and coordinators, and sent weekly to be reviewed and numerically coded by the local Fundação Oswaldo Cruz (FIOCRUZ) research team. Numeric coding was used so translation between Portuguese and English was not necessary for most responses. Coded questionnaires were entered into a database using Epi-Info (Centers for Disease Control and Prevention, 1600 Clifton Road, Georgia, USA). At any point during the study, questionnaires with missing data or incorrect data entry were returned to the field for collection of the missing information.

#### Statistical analysis

Data were analyzed using Stata 11.1 (StataCorp LP, College Station, TX). Individuals who answered “don’t know”, refused to answer, or were missing data for a question had their response excluded in the analysis of that question. For open ended questions, if minor spelling or typographical errors occurred, the appropriate correction was made. Entries that were indecipherable were re-checked with the original survey and counted as missing data if still illegible.

The primary outcome measure, 30-day period prevalence, was defined as the number of participants reporting at least one episode of diarrhoea within the 30 days prior to the interview divided by the total number of respondents interviewed. Thirty day period prevalence was calculated for all survey respondents, and separately for those with and without cisterns. A subgroup analysis was also conducted on children less than five years old to determine the overall 30-day period prevalence and the 30-day period prevalence for those with and without cisterns in this age group. Differences in health outcomes between groups were assessed using Pearson’s Chi-squared tests and two-way t-tests with 95% confidence intervals.

The incidence rate was estimated according to the definition and formula as described by Dohoo et al. (Incidence = number of new cases of disease in a defined time period/ number of people-time units at risk during the time period) [26]. Individuals were asked to report their occurrence of diarrhoea in the previous 30 days, thus 30 days was used as the maximum days at risk per person. Individuals could not be at risk for another episode while experiencing diarrhoea and for one day after the episode. Therefore, the total number of days at risk was calculated by subtracting both the mean number of days sick per month and one day (not at risk following episode) times the number of episodes from the maximum number of days at risk (30 days) (Total days at risk = 30 - [(number of days sick + 1) * number of episodes]).

For the household analysis, households were excluded if they had at least one person in the house who did not respond to the question regarding whether they had diarrhoea in the previous 30 days. Households were analyzed in the following groups: all households, households with cisterns, households without cisterns, and where appropriate, all cases from all households, all cases from households with cisterns, and all cases from households without cisterns. The 30-day period prevalence (at least one person with at least one case in the 30 days prior to the survey) for the household was calculated along with the mean and standard deviation of the percentage of people in the household experiencing diarrhoea at least once during the 30 days prior to being surveyed. Finally, the mean number of diarrhoeal episodes per household and the mean number of household diarrhoeal episodes per person were calculated.

Demographic variables were tested for univariable associations with individual level occurrence of experiencing diarrhoea at least once during the 30 days prior to being surveyed using mixed logistic regression with random effects to account for clustering at the municipality, community, and household level. Individuals were not included in the analysis if community or municipality information was missing or illegible. Continuous variables were assessed for linearity. Age, mother’s education level, and father’s education level were not linear; therefore, they were categorized according to the cut-off points used for census data in Brazil, with the exception of mother and father’s education levels where grade 9 and higher were combined into one group. The latter was done because of the small number of parents who had reached these higher levels of education. Odds ratios and 95% confidence intervals were reported.

#### Ethical approval

Ethical approval was obtained from the Comissão Nacional de Ética em Pesquisa, Brazil and Health Canada’s Research Ethics Board.

## Results

### Response rates and descriptive statistics

A total of 3747 people completed the survey from 789 households, 162 communities and 21 municipalities. This was close to the target sample size of 816 households which was not met due to insufficient time. The response rate was 100% at both the household and individual level (789/789 households and 3747/3747 individuals); the interviewers were local residents from the same municipality as the participants which likely helped to improve participation. Fifty-one people from 12 of these households were not included in the analysis because the household was later determined not to meet the eligibility criteria. Seven people from five of those households were also not included in the analysis because they did not respond to the primary outcome question of whether they had experienced diarrhoea at least once in the 30 days prior to being surveyed. An additional 10 people from three households were not included in the analysis because of data errors. This left a total of 3679 people from 774 households, 160 communities and 21 municipalities to be included in the analysis. Specifically, there were 1863 people from 377 households with cisterns and 1816 people from 397 households without cisterns.

### Individual burden of illness

Of the 3679 respondents, 535 reported experiencing at least one episode of diarrhoea during the 30 days prior to the interview, corresponding to an overall 30-day period prevalence of 14.5% (95% CI 13.4-15.7). Participants from a household with a cistern had a 30-day period prevalence of 11.0% (204/1863; 95% CI 9.5-12.4). Those from a household that did not have a cistern had a significantly higher 30-day period prevalence (Pearson’s Chi-squared; p < 0.001) of 18.2% (331/1816; 95% CI 16.4-20.0). Among children under five years of age, the overall 30-day period prevalence was 21.3% (202/949; 95% CI 18.7-23.9). Children under five years old in households with a cistern had a significantly lower 30-day period prevalence of 15.6% (72/462; 95% CI 12.3-18.9) versus the 26.7% (130/487; 95% CI 22.8-30.6) observed in those children from households without cisterns (Pearson’s Chi-squared; p < 0.001).

The annual incidence rate of diarrhoea was 2.77 episodes per person-year for the entire study group, and 2.14 and 3.43 episodes per person-year for participants from households with and without a cistern, respectively. Among children under five years of age, the overall annual incidence rate was estimated to be 4.00 episodes per person-year. Children under five years old from households with a cistern had an incidence rate of 3.15 episodes per person-year, while those from families without cisterns had a higher rate of 4.83 episodes per person-year.

### Symptomology, duration and health care

The frequency, symptoms, and health care sought for diarrhoea are shown in Table 1. Of the 535 diarrhoeal cases in the entire study population, 11.7% (62/531) reported having blood in their stool and 89.0% (460/517) reported liquid/watery stools. On average, cases had diarrhoea for 3.9 days, with a mean of 1.5 episodes per 30 days, with 36.7% (196/534) of cases reporting more than one episode. Of those who had diarrhoea, 49.1% (260/530) reported taking medication or home therapy. Health care was sought by 42.6% (228/535) of the participants with diarrhoea; 9.5% (51/535) contacted a Community Health Agent, 23.2% (124/535) were referred to a Health Post, 11.4% (61/535) went to a hospital, and 1.1% (6/535) sought other forms of help. There were no significant differences in symptomology, duration of illness, and care sought between individuals in households with or without cisterns.

**Table 1:** Symptoms, duration, number of episodes, treatment, and health care sought among cases of diarrhoea in individuals living in homes with and without cisterns

	**All cases**	**Cases with Cisterns**	**Cases without Cisterns**	**p-value†**
**Symptoms (Number and %)**				
Blood in stool	62/531 (11.7%)	25/202 (12.4%)	37/329 (11.3%)	0.694
Liquid/watery stool	460/517 (89.0%)	178/199 (89.5%)	282/318 (88.7%)	0.786
**Duration (Per month)**				
Mean number of days with diarrhoea and SD	3.9 (n = 534)	3.6 (n = 204)	4.0 (n = 330)	0.183
		SD 2.83	SD 3.26	
**Number of Episodes (Per month)**				
Mean number of diarrhoeal episodes and SD	1.5 (n = 534)	1.6 (n = 204)	1.5 (n = 330)	0.307
		SD 0.96	SD 0.80	
Number and percent of cases with more than one episode	196/534 (36.7%)	76/204 (37.3%)	120/330 (36.4%)	0.836
**Treatment (Number and %)**				
Took medication or home therapy	260/530 (49.1%)	106/202 (52.5%)	154/328 (47.0%)	0.217
**Care Sought (Number and %)**				
None	307/535 (57.4%)	114/204 (55.9%)	193/331 (58.3%)	0.582
Community Health Agent (ACS)	51/535 (9.5%)	22/204 (10.8%)	29/331 (8.8%)	0.439
Referral to Health Post	124/535 (23.2%)	46/204 (22.6%)	78/331 (23.6%)	0.787
Referral to hospital	61/535 (11.4%)	27/204 (13.2%)	34/331 (10.3%)	0.295
Other	6/535 (1.1%)	2/204 (1.0%)	4/331 (1.2%)	0.808

SD: standard deviation.

† Comparison between individuals with and without cisterns using Pearson’s Chi-squared test for differences in symptoms, treatment and health care sought and two-way t-test for differences in means.

### Household burden of illness

An additional five households (one household with a cistern and four households without a cistern) were excluded from the household analysis as they had at least one person in the house that did not respond to the question of whether they had diarrhoea in the previous 30 days, reducing the number of households from 774 to 769 (376 with cisterns and 393 without cisterns). The 30-day period prevalence at the household level was 39.1% (301/769; 95% CI 35.7-42.6) (Table 2). Households with a cistern had a period prevalence of 32.2% (121/376; 95% CI 27.4-36.9), while households without a cistern had a significantly higher (Pearson’s Chi-squared; p < 0.001) period prevalence of 45.8% (180/393; 95% CI 40.9-50.7). During the 30-day study period, households with a cistern had a significantly lower percent of people within the household experiencing diarrhoea at least once, mean number of episodes per household and mean number of diarrhoeal episodes per person compared to households with a cistern (Table 2). Among households that experienced at least one episode of diarrhoea within the 30-day study period, households without a cistern had a significantly higher mean percent of people with diarrhoea per household but there were no significant differences between households with and without cisterns with respect to mean number of diarrhoeal episodes per household or per person within the household (Table 2).

**Table 2:** Diarrhoea during the 30 days prior to being interviewed at the household level for households with and without cisterns, with a subgroup analysis of households with at least one person experiencing one or more episodes of diarrhoea

	**Households**	**Households with Cisterns**	**Households without Cisterns**	**p-value†**	**All cases**	**Household cases with Cisterns**	**Household cases without Cisterns**	**p-value††**
	**(n = 769)**	**(n = 376)**	**(n = 393)**		**(n = 301)**	**(n =121)**	**(n = 180)**	
**Households with at least one person experiencing diarrhoea at least once (Number and %)**								
	301	121 (32.2%)	180 (45.8%)	<0.001	-	-	-	-
	(39.1%)							
**Frequency of people experiencing diarrhoea per household (%)**								
Mean percent of people with diarrhoea per household and SD	15.5%	11.5%	19.3%	<0.001	39.5%	35.8%	42.1%	0.019
		SD 0.20	SD 0.27			SD 0.20	SD 0.24	
**Household episodes (Number)**								
Mean number of diarrhoeal episodes per household and SD	1.05	0.85	1.25	0.004	2.69	2.64	2.72	0.766
		SD 1.74	SD 2.04			SD 2.16	SD 2.25	
Mean number of household diarrhoeal episodes per person and SD	0.23	0.18	0.28	0.001	0.60	0.57	0.61	0.462
		SD 0.37	SD 0.43			SD 0.46	SD 0.46	

SD: standard deviation.

† Comparison between households with and without cisterns using Pearson’s Chi-squared test for differences in prevalence and two-way t-test for differences in means.

†† Comparison between households with and without cisterns among cases using two-way t-test for differences in means.

### Demographic associations

Univariable associations, adjusted with random effects for household, community, and municipality, are shown in Table 3. Twenty-five individuals had either missing or illegible information regarding community, and/or municipality and were unable to be included in the analysis. Children less than five years old were more likely to have experienced diarrhoea in the 30 days prior to the interview compared to all other age groups. This was significant except when children under five were compared to those between the ages of 51–65 years of age (95% CI 0.25-1.03). For every additional family member residing in the home there was a 0.88 increase in the odds of an individual within the home having diarrhoea (95% CI 0.80-0.96). Gender, mother’s and father’s literacy status and education, and any other sources of income (other than self employed farmer) were not significantly associated with diarrhoea.

**Table 3:** Frequency distribution, number of cases and univariable associations with 30-day period prevalence of diarrhoea in respondents from households with and without cisterns in the Agreste Central Region of Pernambuco State, Brazil (n=3654)

**Variable**	**Participants no. (%)**	**Cases no.**	**OR**	**95% CI**
**Gender (n = 3651)**				
Male	1808 (49.5)	262	Referent	
Female	1843 (50.5)	272	0.97	0.77-1.21
**Age (years) (n = 3653)**				
<5	944 (25.8)	201	Referent	
5-10	571 (15.6)	78	0.44	0.31-0.64
11-20	607 (16.6)	58	0.32	0.22-0.47
21-30	651 (17.8)	94	0.48	0.35-0.67
31-40	469 (12.8)	53	0.32	0.21-0.48
41-50	208 (5.7)	30	0.57	0.33-0.96
51-65	124 (3.4)	13	0.50	0.25-1.03
>65	79 (2.2)	7	0.38	0.15-0.98
**Mother’s Literacy (n = 3649)**				
Illiterate	1120 (30.7)	157	Referent	
Literate	2529 (69.3)	377	0.97	0.66-1.43
**Mother’s Education Level if Literate (n = 2477)**				
Grades 1-3	791 (31.9)	119	Referent	
Grades 4-8	1544 (62.3)	230	1.14	0.72-1.81
Grades 9-university	142 (5.7)	22	1.33	0.54-3.30
**Father’s Literacy (n = 3540)**				
Illiterate	1762 (49.8)	263	Referent	
Literate	1778 (50.2)	255	0.84	0.59-1.20
**Father’s Education Level if Literate (n = 1684)**				
Grades 1-3	742 (44.1)	114	Referent	
Grades 4-8	879 (52.2)	118	0.98	0.56-1.69
Grades 9-university	63 (3.7)	13	2.12	0.61-7.32
**Source of Income**† **(n = 3654)**				
Government worker	144 (3.9)	23	1.21	0.53-2.74
Salaried rural worker	160 (4.4)	20	0.83	0.37-1.88
Self employed farmer	2523 (69.1)	315	0.57	0.38-0.85
Sales and service	38 (1.0)	9	1.80	0.46-6.98
Skilled labourer	92 (2.5)	20	1.41	0.52-3.82
Unskilled labourer	255 (7.0)	32	1.09	0.52-2.27
Family assistance	2607 (71.4)	358	0.87	0.60-1.24
Old age pension	543 (14.9)	76	1.00	0.61-1.64
Other	161 (4.4)	29	1.52	0.66-3.50
**Number of People in Household (n = 3654)**	Mean = 5.55		0.88	0.80-0.96

Standard errors corrected using mixed logistic regression with random effects for household, community, and municipality.

OR: odds ratio; CI: 95% confidence interval.

† Respondents could select more than one response.

## Discussion

This study found that the 30-day period prevalence of acute diarrhoea was significantly higher among individuals living in households without cisterns when compared to individuals living in households with cisterns. Additionally, children under five years of age (both with and without cisterns) had a significantly higher period prevalence of diarrhoea than all other age groups except when compared to those between the ages of 51 and 65 years old. In this instance, those under 5 did have a higher period prevalence but the difference was not significant which may have been a result of small sample sizes among the 51 to 65 year old age group. It is interesting to see that in most instances children under five years of age had a significantly higher period prevalence compared to other age groups. A possible explanation for this finding could be that children under five years have not had a chance to acquire immunity to the pathogens found in their drinking water. A significant reduction in 30-day period prevalence of diarrhoea among households with cisterns was also found at the household level.

To the authors’ knowledge, this is the first study to estimate the prevalence of diarrhoea with and without the use of cisterns in a developing country and as such, no direct comparisons with other populations can be made. However, there have been studies that have estimated prevalence and incidence of diarrhoea in developing countries. Burden of illness studies in Cuba, Argentina, and Chile have estimated the 30-day period prevalence of diarrhoea to be between 3.44 and 10.6% [27-29]. Participants from these studies were from a variety of different rural and urban settings, and tended to be older and more educated than the participants in our study. This may help to explain why the 30-day period prevalence was higher (14.5%) in our study.

A literature review of 11 articles on diarrhoeal incidence among children under five years of age in developing countries by the WHO estimated the median incidence for children under five years old in developing countries to be 3.2 episodes per child-year (range = 2.3-6.3 episodes per child year) [30]. One of the studies included in this review was conducted in an urban slum in northeast Brazil and estimated the under five years old incidence of diarrhoea to be 5.25 episodes per child year [31]. These estimates are similar to our 3.15 and 4.83 episodes per child-year for those with and without cisterns, respectively.

The reduction of diarrhoea with the use of cisterns is supported by results from previous trials investigating the relationship between rainwater use and diarrhoea [16,18]. A quasi-experimental design conducted in rural villages in Kenya, although not specifically looking at cisterns, reported that the use of harvested rainwater as a drinking water source, mostly via all available collection vessels (e.g., buckets and barrels), significantly reduced the risk of diarrhoea by 30% [16]. This is consistent with a developed country study in Australia that found no significant difference in gastroenteritis rates between children who consumed treated public mains water compared to those who drank household cistern water [18].

Although these studies and our current findings support the use of cisterns to reduce diarrhoea in developing countries, or not to increase diarrhoea in developed countries, studies investigating the microbiological and chemical quality of rainwater from cisterns have found conflicting results. In Bermuda, samples from rainwater tanks have been found to be highly contaminated, with over 90% of samples taken containing more than 10 CFU/100 ml of total coliforms [32]. However, these results may not have been representative of rainwater tanks in developing countries as the trial was conducted after a hurricane. This may have increased dust and dirt on the rooftops from which the rainwater was collected. In Australia, samples from rainwater cisterns contained *E. coli*[33] and heavy metals from sediments [34]. There seems to be a disconnect between reductions in diarrhoea and water quality with respect to rainwater from cisterns. Ahmed *et al.* (2008) suggest that fecal indicators used in many microbiological water quality studies may not be adequate to assess the microbiological quality of rainwater because of poor correlation between fecal indicators and potential pathogens. In addition, environmental organisms have been found to contribute to the bacterial load in rooftop rainwater in Australia and may be important in processes within the cistern tank that may have a beneficial impact on the microbiological quality of water in the cistern [35]. Also, these water quality studies were conducted in developed countries using relatively small sample sizes and may not be applicable in rural settings in developing countries. Further work should be conducted to explore the relationship between the use of rainwater from cisterns, reductions in diarrhoea and corresponding microbiological water quality. It is possible that, in the semi-arid region of Brazil, the use of cisterns in place of contaminated surface water would still be beneficial even if there was some contamination of the water from the rooftop, and that this contamination could be reduced using point of use disinfectants, filtering, boiling, or educational methods.

Not all diarrhoea is due to waterborne causes, and therefore, it is not expected that all diarrhoea in northeast Brazil could be prevented or reduced with the use of a household cistern. However, there is evidence to support that reducing waterborne diarrhoeal episodes will have an impact on the reduction of overall diarrhoeal episodes, as the WHO estimates that 88% of the estimated 4.4 billion cases of diarrhoea are attributable to unsafe water, inadequate sanitation and hygiene [4].

Symptomology, duration of diarrhoea and type of health care sought was not significantly different between those with and without cisterns. Hence, although the number of episodes appears to be lower among individuals with cisterns, it does not affect the length and severity of illness when it does occur, nor the types of care sought for diarrhoea. We would expect that people with more severe symptoms for a longer duration would seek medical care more often than those with mild diarrhoea for a short duration. The lack of difference may indicate that these two groups are experiencing diarrhoea caused by similar pathogens, albeit less frequently in the cistern group. Future studies should be implemented to explore this hypothesis; perhaps by investigating the effect of cistern use on diarrhoea while additionally collecting faecal samples to determine the agent responsible. This could also provide further evidence linking the episodes of diarrhoea with a waterborne pathogen.

Gender, mother’s and father’s literacy status and education level, along with several sources of income were not found to be independently significantly associated with diarrhoea. Previous studies conducted in developing countries have also found gender [9,28,29], mother’s education [16] and mother’s and father’s literacy status [36] not to be risk factors for diarrhoea. In this study, reasons for this could be that our population was relatively uniform in that approximately 70% of mothers and half of fathers were literate, with the majority of the parents who were literate having low levels of education. Also, the majority of household incomes came from self employed farming and/or family assistance. Therefore, differences may not have been found in literacy levels, education and sources of income other than self employed farming or family assistance due to a lack of statistical power.

There are a few limitations to this study, one being that we did not randomly select our households. Randomization was attempted but unsuccessful because there were inconsistencies between the list of cisterns that had been built and the actual number on the cistern at the households. While the total number of cisterns built in each community was known, the authors were not able to determine the specific location and household of cisterns within each community and were therefore unable to create a sampling frame. Controls were selected by interviewers based on proximity to households with cisterns, as the risk of diarrhoea can be dependent on location. Having interviewers select controls could be a source of selection bias however, this approach ensured that those without cisterns were similarly distributed among communities and municipalities as those with cisterns. In addition, households with and without cisterns were selected within each community and municipality in proportion to the number of cisterns that had been built in that particular community and municipality, with a minimum of four households (two with cisterns and two without) selected per community. Every household member within each household was interviewed and included in the study, and a large sample size was achieved. The systematic sampling method, large sample size and a 100% response rate was thought to capture a study group that was representative of the study population of interest.

Secondly, we were unable to compare the demographic characteristics or typical water treatment practices of our study population with the population from which the sample was taken. This is because census information from Agreste Central Region of Pernambuco State is not consistently collected for all municipalities. Due to the large sample size used for this study and the inclusion of all the qualifying municipalities, participants were thought to represent the population who qualify for a cistern from the P1MC. Our study may not be representative of all people who live in rural households as they could have different water needs, income, and socio-economic statuses. It is also expected that participants were on average younger than those in the general population due to the selection criteria of households requiring at least one child less than five years old.

This study used a 30-day recall period so as to be comparable with burden of illness studies in developing countries [27-29]. There is a potential for recall bias when using a longer recall period, however, population surveys in an Argentine community and a Metropolitan region in Chile have compared 7-day, 15-day and 30-day recall periods for acute gastrointestinal illness and found between 1.7-5.4 times higher annual incidence rates compared to the 30-day recall period. These results suggest an underestimate of the true burden of disease when using a longer recall period. The potential for recall bias should be further explored in future studies, in particular, in the Agreste Central Region of Pernambuco State, Brazil. Finally, it was difficult to determine if families used only rooftop rainwater to fill their cistern. When families receive their cisterns they are informed not to fill it with outside water, such as water that is trucked in from other areas, as it may not be free of contaminants. Despite this instruction, outside water may have been used, although the extent to which this may have occurred was not assessed. Therefore this study may have underestimated the association between the 30-day period prevalence of diarrhoea and the use of cisterns that are only filled with rainwater.

Our study did not analyze additional risk factors for diarrhoea such as presence of a latrine in the home, regular use of soap, point of use disinfectants and practices, whether water was boiled and demographic variables that have been previously shown in other populations to impact the rate of diarrhoea [2,15,16]. Future studies should take into account these risk factors as potential confounders. Our study duration was only 30 days and conducted shortly after the rainy season. This time period was chosen because higher rates of diarrhoea are typically seen and cisterns should have had adequate amounts of water stored. Future studies should estimate the prevalence of diarrhoea in the dry season, or for an entire year, to determine if these differences between groups occur year round.

## Conclusions

Our results indicate that there was a lower prevalence of diarrhoea among individuals whose household had a rainwater cistern compared to individuals whose household did not have a cistern, particularly among young children. The results also suggest that there is no difference in symptomology, duration of illness and health care sought between people with cisterns and those without. The lower prevalence among individuals with cisterns illustrates the importance of improving water access through initiatives such as the P1MC in northeast Brazil. Future studies should further investigate this association while accounting for additional risk and preventative factors that may be associated with diarrhoea among the study population.

## Competing interests

The authors declare that they have no competing interests.

## Authors’ contributions

JAF, AMB, CFL, MKT, TML, EP and AMC designed the study. The study was coordinated by JAF, AMB, ES, EP and AMC. AMB, CFL, ES and AMC collected the data. Analysis and interpretation of the data was conducted by PBM, JAF, JMS, AQJ, CFL, ES, and TML. The manuscript was drafted by PBM, JAF, JMS and AQJ. All authors critically reviewed and have given final approval of the manuscript.

## Pre-publication history

The pre-publication history for this paper can be accessed here:

http://www.biomedcentral.com/1471-2334/13/65/prepub
